# What has driven the evolution of multiple cone classes in visual systems: object contrast enhancement or light flicker elimination?

**DOI:** 10.1186/1741-7007-11-77

**Published:** 2013-07-04

**Authors:** Shai Sabbah, Craig W Hawryshyn

**Affiliations:** 1 Department of Biology, Queen’s University, Kingston, Ontario K7L 3N6, Canada; 2 Centre for Neuroscience Studies, Queen’s University, Kingston, Ontario K7L 3N6, Canada

**Keywords:** Contrast hypothesis, Cone photoreceptors, Critical fusion frequency, Temporal contrast sensitivity, Opponent mechanisms, Color vision, Retina, Fish

## Abstract

**Background:**

Two competing theories have been advanced to explain the evolution of multiple cone classes in vertebrate eyes. These two theories have important, but different, implications for our understanding of the design and tuning of vertebrate visual systems. The ‘contrast theory’ proposes that multiple cone classes evolved in shallow-water fish to maximize the visual contrast of objects against diverse backgrounds. The competing ‘flicker theory’ states that multiple cone classes evolved to eliminate the light flicker inherent in shallow-water environments through antagonistic neural interactions, thereby enhancing object detection. However, the selective pressures that have driven the evolution of multiple cone classes remain largely obscure.

**Results:**

We show that two critical assumptions of the flicker theory are violated. We found that the amplitude and temporal frequency of flicker vary over the visible spectrum, precluding its cancellation by simple antagonistic interactions between the output signals of cones. Moreover, we found that the temporal frequency of flicker matches the frequency where sensitivity is maximal in a wide range of fish taxa, suggesting that the flicker may actually enhance the detection of objects. Finally, using modeling of the chromatic contrast between fish pattern and background under flickering illumination, we found that the spectral sensitivity of cones in a cichlid focal species is optimally tuned to maximize the visual contrast between fish pattern and background, instead of to produce a flicker-free visual signal.

**Conclusions:**

The violation of its two critical assumptions substantially undermines support for the flicker theory as originally formulated. While this alone does not support the contrast theory, comparison of the contrast and flicker theories revealed that the visual system of our focal species was tuned as predicted by the contrast theory rather than by the flicker theory (or by some combination of the two). Thus, these findings challenge key assumptions of the flicker theory, leaving the contrast theory as the most parsimonious and tenable account of the evolution of multiple cone classes.

## Background

Multiple spectral classes of cones are found in the visual system of many vertebrates [1]. Comparison of the outputs of different cone classes enables color vision. Multiple cone classes appeared very early in vertebrate evolution, at least 540 MYA (million years ago) and perhaps as early as 700 MYA, prior to the separation of the jawed (Gnathostomata) and jawless (Agnatha) vertebrate lineages (approximately 485 MYA) [2,3]. This is based on the presence of five classes of cone-like photoreceptors in the jawless Southern Hemisphere lamprey, *Geotria australis*[4-6], and three cone classes in the jawed cartilaginous fishes (Chondrichthyes) [7-9]. Additionally, cone opsins have been suggested to evolve prior to rod opsins [10], indicating that photopic (bright light) vision preceded scotopic (dim light) vision, and suggesting that these early vertebrates occupied brightly-lit shallow-water environments [11]. However, although the evolution of visual pigments has been studied extensively [1,4,6,10,12-20], the selective pressures that have driven the evolution of multiple cone classes in the eyes of vertebrates remain largely obscure.

Two competing theories have been advanced to explain the evolution of multiple cone classes; both assumed that vision in ancestral vertebrates utilized multiple cone photoreceptor classes, with color vision evolving only later as a byproduct. The ‘contrast theory’ of Munz and McFarland and McFarland and Munz [13,14] proposed that multiple cone classes evolved in shallow-water fish to maximize the visual contrast between objects and their background. Indeed, a single visual pigment (either rod or cone) may suffice to maximize the visual contrast between a given object and background. However, the need to maximize contrast between diverse objects and backgrounds of varying brightness and spectral characteristics was suggested to favor the appearance of multiple cone classes. The competing ‘flicker theory’ presented by Maximov [21] proposed that multiple cone classes have evolved to allow elimination of the flicker (fluctuation in light intensity) produced by variation in the refraction of sunlight at the water surface [22-25]. It was argued that subtraction of the output of one cone class from another through antagonistic (opponent) neural interactions would filter out the light flicker, yielding a flicker-free representation of the visual scene and enhancing object detection. The flicker theory has received relatively little attention; however, it has remained a competitor of the contrast theory, leaving the forces that have driven the evolution of multiple cone classes an open question.

Both the contrast and flicker theories assume the presence of at least two cone classes that differ in spectral tuning. The flicker theory rests on three additional assumptions, one of which is the presence of antagonistic interactions between the output signals of the available cone classes. This assumption receives support from the presence of color-opponent horizontal cells [26,27] and the concentrically-antagonistic center-surround organization in retinal bipolar [28,29] and ganglion cells [30] in lower vertebrates. At least some of these color opponent mechanisms were probably present in early vertebrates that are represented today by the jawless lampreys [31-33]. However, two other critical assumptions of the flicker theory have so far not been seriously examined. First, it is assumed that ‘the [light] fluctuations are colorless, that is, the intensity of light changes synchronously in different parts of the spectrum’ [21]. Consequently, despite the strong fluctuations in light over the entire spectrum, the ratio of light intensities in two different parts of a spectrum would remain constant, and would depend only on the spectral properties of the viewed object. Second, the flicker theory assumes that ‘the significant flicker of illumination inherent in the shallow-water environment complicated the visual process in the achromatic case, in particular preventing early detection of enemies’ [21]. Thus, because light flicker would impair object detection, selection would favor removal of light flicker from the processed visual signal. These critical assumptions of the flicker theory have never been tested.

In this report, we evaluated the relative merits of the contrast and flicker theories. We first focused on the two largely untested assumptions of the flicker theory, and then asked whether the predictions of the two theories regarding the spectral tuning of cone pigments are supported by the evidence. We found that the amplitude and temporal frequency of light flicker are wavelength dependent and that the flicker may actually enhance the detection of objects, thus violating critical assumptions of the flicker theory. While this alone does not support the contrast theory, comparison of the contrast and flicker theories by means of chromatic contrast modeling under flickering illumination revealed that the spectral tuning of cone pigments of a focal cichlid species produced a large chromatic contrast between background and the body pattern of fish, and did not allow elimination of temporal fluctuations in the visual signal. This suggests that the visual system of the focal species is tuned as predicted by the contrast theory rather than by the flicker theory (or by some combination of the two).

## Results and discussion

### Amplitude and temporal frequency of flicker are wavelength dependent

**Figure 1:**
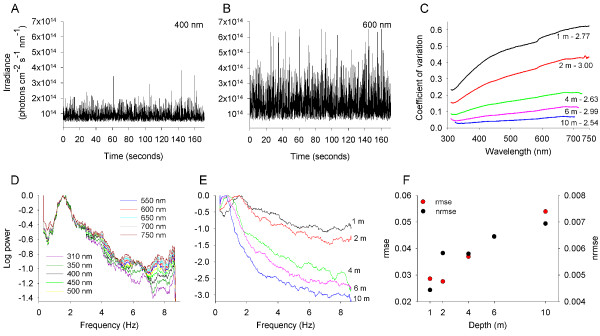
**Amplitude and temporal frequency of the light flicker of downward irradiance are wavelength dependent. (A,B)** Examples of light flicker time series of downward irradiance at 1 m depth and light wavelengths of 400 and 600 nm. Each time series constitutes of 3,000 measurements acquired over 173 s. The amplitude of the light flicker at 600 nm is larger than at 400 nm. **(C)** The amplitude (estimated as the coefficient of variation) of light flicker of downward irradiance decreased with growing water depth (across the 1 to 10 m depth range), and increased monotonically toward longer light wavelengths. The ratio between the amplitude at the longest and shortest wavelengths was calculated for each depth. This ratio did not vary considerably across depths, and ranged between 2.5 and 3.0 (presented next to each spectrum). **(D)** The frequency distribution of the flicker at a depth of 1 m differed across the light spectrum. For clear graphical presentation, the power spectrum of light flicker, normalized to the dominant frequency (1.54 Hz), is presented for different wavelengths at 50 nm intervals. See Additional file 1A-D for the frequency distribution of flicker at 2, 4, 6, and 10 m depth. **(E)** The frequency distribution of light flicker at 500 nm differed across water depths, with the dominant frequency (1 m, 1.54 Hz; 2 m, 1.54 Hz; 4 m, 0.83 Hz; 6 m, 0.80 Hz; 10 m, 0.67 Hz) and the relative power at high frequencies decreasing with growing depth. **(F)** The wavelength dependence of light flicker became weaker with growing depth. Wavelength dependence was assessed as the reciprocal of the root mean square error (RMSE) and the normalized RMSE (NRMSE) between the power distribution at 500 and 550 nm.

The first critical assumption of the flicker theory is that ‘the [light] fluctuations are colorless’ [21], that is, the amplitude of the flicker and the distribution of its power across temporal frequencies are similar across the light spectrum. Only in this special case would the simple subtraction of outputs of different cone classes through color opponent channels produce a flicker-free visual signal, as posited in the original theory. To study the characteristics of underwater light flicker, we measured at high temporal resolution the downward and sideward irradiance from 310 to 750 nm at a range of water depths. Figure 1A,B illustrates light flicker time series of downward irradiance at 1 m depth and light wavelengths of 400 and 600 nm. To estimate the amplitude of the flicker, we calculated the coefficient of variation (CV) of the time series of downward irradiance at each light wavelength. CV decreased with increasing water depth. However, consistent with past studies [22,34,35], CV increased monotonically at all depths toward longer light wavelengths, with long wavelengths 2.5 to 3 times more variable than short wavelengths (Figure 1C). To study the temporal frequency of the light flicker, we calculated the power spectrum of the downward irradiance time series at each light wavelength. The power distribution of the flicker across frequencies varied with light wavelength (Figure 1D; Additional file 1A-D), consistent with a past study [34]. Additionally, the frequency distribution of flicker varied with water depth, and the dominant frequency of flicker decreased with increasing depth (Figure 1E). The wavelength dependence of the power distribution of flicker also varied with water depth, such that the wavelength dependence of the power distribution became weaker with increasing depth (Figure 1F). These findings are consistent with variation observed in the wavelength dependence of the power distribution of light flicker across different viewing orientations [34]. (See Additional file 2 for similar analysis for sideward irradiance.) Light flicker is produced by the focusing and defocusing of sunlight rays refracted at the water surface [22,24]. The wavelength dependence of the amplitude and temporal frequency of the flicker arises largely from variation in the scattering of light across the spectrum. Scattering at short wavelengths by molecules and small particles in the atmosphere and water is generally more pronounced than at long wavelengths. This causes short-wavelength light to be more diffused and consequently less affected by the wave-focusing phenomenon than long-wavelength light. Therefore, our results demonstrate that the amplitude of the flicker and the distribution of its power across temporal frequencies vary across the light spectrum, violating the flicker theory’s first assumption.

### Subtraction of cone outputs through opponent channels does not produce a flicker-free visual signal

We examined the effect of the wavelength dependence of light flicker on the output of cones and on the capacity of a simple subtraction of cone outputs to eliminate the flicker from the processed signal. To this end, we calculated the cone output (estimated by the quantum catch of cone pigments) when viewing an achromatic target (reflectance = 50% across the spectrum) under flickering illumination in a focal species, *Metriaclima zebra* (a Lake Malawi cichlid). The cone pigment complement found in adult *M. zebra* includes the SWS1 (368 nm), Rh2b (484 nm), and Rh2a (523 nm) pigments (wavelength values represent the wavelength of maximum absorbance of cones, λ_max_) [36]. The target viewed was assumed to be illuminated by flickering sideward irradiance at a depth of 1 m, and cones were assumed to be adapted to the mean sideward irradiance at the same depth. (See the Methods section for detailed description of how cone and opponent channel outputs were calculated).

**Figure 2:**
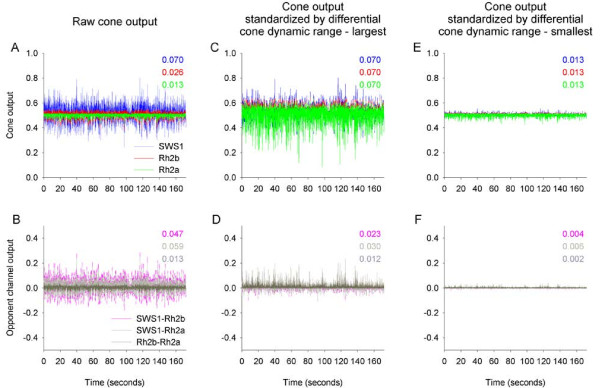
**Neural opponent interactions between cone outputs do not produce flicker-free visual signal. (A)** Normalized output of the three real cones in adult *Metriaclima zebra* in response to light reflected off an achromatic target (50% reflectance) that was illuminated with flickering illumination. Cone output varied considerably over time, with the variation in cone output (estimated as the standard deviation over time) highest for SWS1, lower for Rh2b, and lowest for Rh2a. **(B)** Output of putative opponent channels (SWS1-Rh2b, SWS1-Rh2a, and Rh2b-Rh2a) varied over time, showing variation comparable to that in cone output. **(C)** Cone dynamic range adjusted such that the over time variation in cone output was similar for all cone classes and equaled that of SWS1 that exhibited the largest variation. **(D)** Following the dynamic range adjustment, the variation in opponent channels’ output decreased only slightly, and did not produce flicker-free visual signals. **(E)** Cone dynamic range adjusted such that the over time variation in cone output was similar for all cone classes and equaled to that of Rh2a that exhibited the smallest variation. **(F)** Following this dynamic range adjustment, the variation in opponent channels’ output decreased by an order of magnitude, producing a visual signal that is almost flicker-free. Colored values indicate the variation in the output of each of the cones and opponent channels.

Cone output varied considerably over time under flickering illumination, with the variation in cone output (estimated as the standard deviation over time) decreasing when moving from SWS1 (0.070), through Rh2b (0.026), and to Rh2a (0.013) (cone output is unitless because it is normalized by the adapting light) (Figure 2A). The output among the three cones varied because long wavelength pigments such as Rh2b and Rh2a show broad sensitivity functions, with both the α- and β-absorption bands included in the 300 to 800 nm spectrum that might be used for vision. By sampling the light flicker across the spectrum, the broad sensitivity functions act to reduce the variation of cone output produced under wavelength-dependent flickering illumination. In contrast, short wavelength pigments such as SWS1 show narrow sensitivity functions, with only the α-absorption band included in the 300 to 800 nm spectrum. These narrow sensitivity functions act to increase the variation in cone output.

The configuration of color opponent channels in *M. zebra* is currently unknown. Thus, we modeled three possible opponent channels, that is, SWS1-Rh2b, SWS1-Rh2a, and Rh2b-Rh2a. Output of opponent channels subjected to flickering illumination varied over time, showing variation largely comparable to that in the output of cones (SWS1-Rh2b, 0.047; SWS1-Rh2a, 0.059; and Rh2b-Rh2a, 0.013) (Figure 2B). Therefore, contrary to the prediction of the flicker theory, simple subtraction of the output of one cone class from that of another through opponent interactions would not produce flicker-free output signal.

Certain modifications to the flicker theory would potentially allow the intended generation of flicker-free visual signals. An obvious way to generate a flicker-free visual signal would be to route the cone output through a low pass filter. This would attenuate any high frequency components in the signal, possibly producing flicker-free opponent channel signals. Such filtration, however, would inevitably reduce the temporal resolution by which the animal is sampling the environment, compromising the animal’s ability to detecting fast moving and changing stimuli, and reacting to them. Furthermore, considering the wavelength dependence of the power distribution of flicker (discussed above), even the low frequency components that are allowed to pass the filter would differ slightly between different cone classes; that is, even opponent channels that only use the low frequency components would not produce a flicker-free signal. Therefore, the use of low pass filtration for generating flicker-free visual signals comes with high cost, and is unlikely to provide a selective advantage.

Adjusting the dynamic range of cones would also potentially allow generation of flicker-free opponent channel output. For example, if the dynamic range of cones were to vary such that variation in cone output across the three cone classes was equalized over time, then subtraction of cone output through opponent channels would potentially produce a flicker-free signal. To test this possibility, we artificially adjusted the dynamic range of cones so that the variation over time in cone output was similar for all cone classes and equaled that of the SWS1 cone that exhibited the largest variation (Figure 2C). Following the dynamic range adjustment, the variation in opponent channels’ output decreased only slightly, and did not produce flicker-free visual signals (SWS1-Rh2b, 0.023; SWS1-Rh2a, 0.030; and Rh2b-Rh2a, 0.012) (Figure 2D). Additionally, such differential adjustment of the dynamic range of cones would reduce the resolution in which the incident color signals (radiance) that vary in intensity are being sampled, potentially compromising the discrimination between targets of relatively close spectral reflectance characteristics. We also artificially adjusted the dynamic range of cones such that the variation in cone output over time was similar for all cone classes, but now, equaled to that of the Rh2a cone that exhibited the smallest variation (Figure 2E). Following this second dynamic range adjustment, the variation in opponent channels’ output decreased by an order of magnitude, producing a visual signal that is almost flicker-free (SWS1-Rh2b, 0.004; SWS1-Rh2a, 0.006; and Rh2b-Rh2a, 0.002) (Figure 2F). Thus, such a mechanism may theoretically generate a flicker-free output of opponent channels. Note however, that such differential adjustment of the dynamic range of cones to span the dynamic range of the cone that shows the narrowest dynamic range would result in failure to sample many incident color signals that fall outside of this narrow dynamic range. This would substantially compromise the detection of targets of various spectral reflectance characteristics, which are illuminated by a range of irradiance levels. Therefore, this possibility is also highly unlikely to have a selective advantage.

Nevertheless, the implementation of complex antagonistic interactions between cone classes (for example, SWS1 + Rh2b - Rh2a), may potentially allow the intended generation of flicker-free visual signals. However, as our light flicker measurements show, the dominant frequency, the frequency distribution of flicker, and the wavelength dependence of the power distribution of flicker all vary with water depth. This suggests that a fixed, ‘wired’ compensation for the wavelength dependence of flicker would fail when an animal moves in the water column or views objects at different lines of sight; behaviors that are common to many fish. In summary, contrary to the prediction of the flicker theory, simple subtraction of the output of one cone class from that of another through opponent interactions would not produce a flicker-free output signal. Moreover, neither fixed low pass filtration nor adjustment of the dynamic range of cones would likely to be favored. Thus, although there might be a mechanism by which flicker-free visual signals would be generated under flickering illumination, the likelihood of such a possibility is low, and the likelihood that such a possibility would be favored either by natural or sexual selection is even lower.

### Temporal frequency of light flicker matches the frequency where maximum contrast sensitivity in fish is attained

A second assumption of the flicker theory is that flicker interferes with object detection. However, by generating periodic changes in the retinal image, flicker may enhance perception [37] and detection of coarse (low spatial frequency) patterns [38]. The flicker is analogous to the flashing of an artificial light, such as a turn signal on a car; it is visually prominent because of the extreme brightness change, and possibly because of an unknown visual alertness system. This enhancement of object appearance would be most efficient if the temporal frequency of the flicker matched the frequency where maximum contrast sensitivity (F_max_) is attained [35,39,40]. Unfortunately, to date, the complete temporal contrast sensitivity function has been determined for only one fish species, the goldfish (*Carassius auratus*) [41], while the temporal contrast sensitivity of fish has often been studied through the measurement of the critical fusion frequency (CFF), the frequency at which temporally modulated light stimuli appear to fuse and have constant brightness. The temporal contrast sensitivity function varies with the adaptation state of the eye, the mean intensity of the modulated light stimulus, the visual angle subtended by the stimulus beam, and the temperature. Nevertheless, it still takes on a rather simple and easily modeled function [42-44], where F_max_ typically ranges between 10% and 20% of CFF [41,45-48]. Thus, to accommodate comparison between the frequency of wave-induced light flicker and the F_max_ of various fish species, as a first-order approximation, F_max_ was estimated as 15% of CFF, and then as either 10% or 20% of CFF, covering the possible realistic range of relationships between F_max_ and CFF.

**Figure 3:**
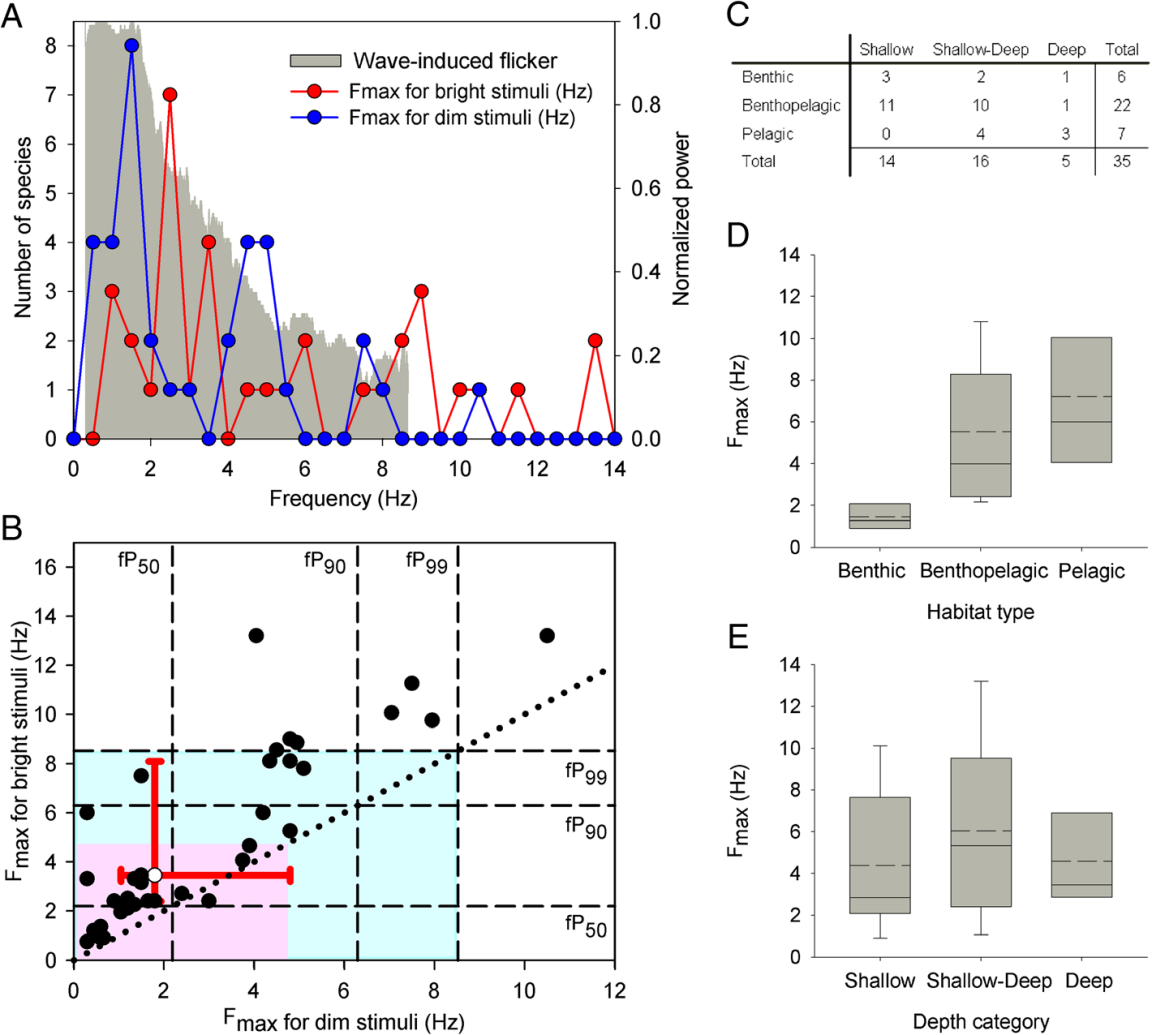
**Light flicker can enhance the detection of underwater objects. (A)** Comparison between the frequency of light flicker in downward irradiance and the F_max_ of fish for dim and bright stimuli. The distribution of F_max_ values across frequency corresponded well to the power spectrum of flicker across depths. Depicted power spectrum (gray shaded area) represents the envelope of flicker power across the 1 m and 10 m depth range. **(B)** Comparison between the cumulative power of the flicker and F_max_ for dim and bright stimuli (closed circles). The indices fP_50_, fP_90_, and fP_99_ stand for the frequencies that correspond to 50, 90, and 99 percent of the cumulative power of flicker across frequencies, averaged across the light spectrum. Vertical and horizontal dashed lines represent the fP_50_, fP_90_, and fP_99_ values at 1 m depth. Gray and pink shaded areas represent the frequency range enclosed by the 99% cumulative power bounds at 1 m and 10 m depth, respectively. (See Additional file 1E-G for the cumulative power indices as a function of light wavelength.) F_max_ data points typically fell above the identity (dotted) line, indicating larger F_max_ values for bright stimuli. The median F_max_ equaled 1.8 and 3.5 Hz, for dim and bright stimuli, respectively (open circle; bidirectional red error bars represent the 25th and 75th percentiles). **(C)** Summary of the number of species inhabiting the various habitats and depths examined. **(D,E)** Association between the F_max_ of fish for bright stimuli and the habitat type (D) and water depth (E). Box specifications: mean (dashed), median (solid), 25th and 75th percentiles; whiskers: 10th and 90th percentiles. For consistency, all analyses included only fish species for which F_max_ data were available for both dim/natural and bright light stimuli (75% of cases), and were obtained under dark adaptation (80% of eligible pairs; *n* = 35); different F_max_ reports for a given species were averaged.

We compiled CFF data for 47 fish species, representing 41 genera, 34 families, and 14 orders, including both bony and cartilaginous fishes. See Additional file 3 for compilation of CFF data and F_max_ estimates with reference to the light adaptation regime and temperature used during experiments, as well as the habitat and depth distribution of each species. F_max_ varied with stimulus intensity, light adaptation regime, and temperature. Nonetheless, with F_max_ estimated as 15% of CFF, the distribution of F_max_ values across frequency corresponded well to the power spectrum of the light flicker, being highest at the dominant frequency of the flicker (Figure 3A). To support our findings further, we calculated the cumulative power of the flicker across temporal frequencies, which, unlike the dominant frequency of the flicker, is rather robust to variation in surface wave conditions. The median F_max_ for dim stimuli, 1.8 Hz, matched the frequency below which close to 50% of the cumulative power of flicker was found at 1 m depth. The median F_max_ for bright stimuli, 3.5 Hz, matched the frequency below which between 50% and 90% of the cumulative power of flicker was found at 1 m depth (Figure 3B). The relative power at high frequencies decreased with increasing depth. Yet, the median F_max_ for both dim and bright stimuli matched the frequency below which 99% of the cumulative power was found at 10 m depth, for which the relative power at high frequencies was the lowest (Figure 3B; Additional file 1E-G). A similar trend was observed when F_max_ was estimated as either 10% or 20% of CFF (Additional file 4). Therefore, these findings suggest that light flicker may enhance the detection by fish of underwater objects under a range of light intensities and water depths, violating the flicker theory’s second assumption.

Note that the correspondence between the F_max_ of fish and the frequency of flicker might potentially break if the fish species included in the analysis were to change. This is especially important if F_max_ varied across environmental categories such as habitat type and depth. Species were classified based on their habitat type as being either ‘benthic’, ‘benthopelagic’, or ‘pelagic’ species. Species were also classified as inhabiting either ‘deep’ (typically found in depths >30 m), ‘shallow’ (typically found in depths <30 m), or both deep and shallow (‘shallow-deep’) habitats. As for the comparison between F_max_ and flicker frequency, the analysis included all 35 fish species for which F_max_ data were available for both dim/natural and bright light stimuli (75% of cases) and were obtained under dark adaptation (80% of eligible pairs). The percentage of species inhabiting shallow (40%) and shallow-deep (46%) habitats was similar, whereas the percentage of strictly deep-water species was lower (14%). Moreover, the percentage of benthopelagic species (63%) was higher than those of either benthic (17%) or pelagic (20%) species (Figure 3C). Thus, if F_max_ differs between habitat or depth categories, this may bias the comparison between F_max_ and flicker frequency. To test this possibility, we examined the effect of water depth and habitat type on the F_max_ of fish. F_max_ for bright stimuli differed significantly across habitat types (randomization test (RT), df = 2, *N* = 35, *P* = 0.0098) (Figure 3D). *Post-hoc* analysis revealed that F_max_ in benthic species was significantly lower than in either benthopelagic (*P* = 0.0095) or pelagic (*P* = 0.0012) species; F_max_ in benthopelagic and pelagic species did not differ significantly (*P* = 0.272). Moreover, F_max_ for bright stimuli did not differ significantly across depth categories (RT, df = 2, *N* = 35, *P* = 0.4472) (Figure 3E). F_max_ did not differ significantly across depth categories also when fish from the ‘shallow-deep’ depth category were pooled with those from the ‘shallow’ category (RT, df = 1, *N* = 35, *P* = 0.6676) or with the ‘deep’ category (RT, df = 1, *N* = 35, *P* = 0.3231). Therefore, F_max_ of fish does not vary with habitat depth; however, F_max_ of benthic species is lower than in the other habitats. Consequently, if benthic species, that represent only 17% of the species analyzed, were to be represented better in the analysis, this would shift the distribution (and median) of F_max_ values toward lower frequencies. Interestingly, inspection of Figure 3A,B reveals that this would actually improve the correspondence between F_max_ of fish and the frequency of flicker, further supporting our conclusions regarding a correspondence between the F_max_ of fish and flicker frequency.

### Contrast theory versus flicker theory: a comparative analysis

The violation of its two critical assumptions substantially undermines support for the flicker theory as originally formulated. However, this alone does not support the contrast theory. Interestingly, considering the wavelength dependence of the light flicker, the two competing theories would predict opposite evolutionary pathways. Using multiple cone classes to increase the contrast of objects would have favored cone classes whose peak sensitivities are far apart. By contrast, using multiple cone classes to eliminate the light flicker would have favored cone classes whose peak sensitivities are the closest possible to each other. Nonetheless, providing conclusive evidence for the dominance of a given evolutionary pathway would require comparing the specific spectral locations of cone pigments of every fish species to the spectrum of ambient light under various conditions and for various behavioral contexts. Such a task is clearly impossible. Here instead, we chose to use a case study, a Lake Malawi cichlid, to assess whether the spectral location of cone pigments has been shaped as predicted by the contrast theory or by the flicker theory, or by both.

For each opponent channel, we calculated responses to a range of naturally occurring body color patterns of fish (measured as diffused spectral reflectance) and the backgrounds against which the fish might be viewed under flickering illumination. The difference between these responses (Δ*C*) is a measure of the chromatic contrast of the particular opponent channel under these specific conditions. The model assigned spectral sensitivities to the cones based on the known characteristics of the cone pigments in this fish. The cichlid *Metriaclima zebra* belongs to the rock-dwelling Mbuna clade. In these species, males defend a territory in between rocks and perform elaborate displays to approaching females against the background of a vertical rock. Thus, following the contrast theory, the visual system would be designed to ensure large Δ*C* magnitude between the body patterns of males and the background of rocks. However, following the flicker theory, the visual system would be structured to ensure small Δ*C* variation under flickering illumination. The prediction regarding the contrast theory follows from the notion that the spectral location of visual pigments has evolved to maximize the chromatic contrast between the color pattern of males and their background. However, at least another possibility exists. That is, that the spectral location of visual pigments in females has been shaped by natural selection, and that males evolved body color patterns to maximize the chromatic contrast against their background, under those pre-existing visual system characteristics of females (‘sensory bias’ hypothesis) [49]. The specific selection forces that have driven the spectral diversification of visual pigments in Lake Malawi cichlids are currently largely unknown [50].

Δ*C* was calculated for two putative types of opponent channels: (i) a channel that compares the outputs of one single cone and one double cone, and (ii) a channel that compares the outputs of two double cones. Lake Malawi cichlids typically display three cone pigments (but see [36,51]), with short-wavelength sensitive pigments (typically λ_max_ <456 nm; SWS1, SWS2b, and SWS2a) occupying single cones, and longer-wavelength sensitive pigments (typically 456 < λ_max_ < 560 nm; Rh2b, Rh2a*,* and LWS) occupying double cones [18,52-54]. Accordingly, λ_max_ of modeled pigments in single cones ranged between 365 and 455 nm (every 5 nm), whereas λ_max_ of modeled pigments in double cones ranged between 460 and 560 nm (every 5 nm). For each modeled pigment pair, Δ*C* was calculated for every single combination of fish body pattern (*n* = 87) and rock background (*n* = 8). Thereafter, we determined the pigment pairs that produced the largest Δ*C* magnitude (‘maxΔ*C*mag’; estimated as the time average Δ*C*) and the smallest Δ*C* variation (‘minΔ*C*var’; estimated as the standard deviation in Δ*C* over time) over 100 consecutive high-temporal resolution sideward irradiance measurements (total duration = 6 s). The larger the Δ*C* magnitude, the larger are the chances that the chromatic contrast between object and background would exceed threshold (as soon as Δ*C* exceeds the threshold, objects can be detected with nearly 100% probability). The smaller the Δ*C* variation, the more efficient is the elimination of flicker from the output of opponent channels. This analysis was repeated for the two most extreme water depths examined in this study, 1 m and 10 m. (See Methods for detailed description of the modeling procedure and Additional file 5 for data used for modeling.)

**Figure 4:**
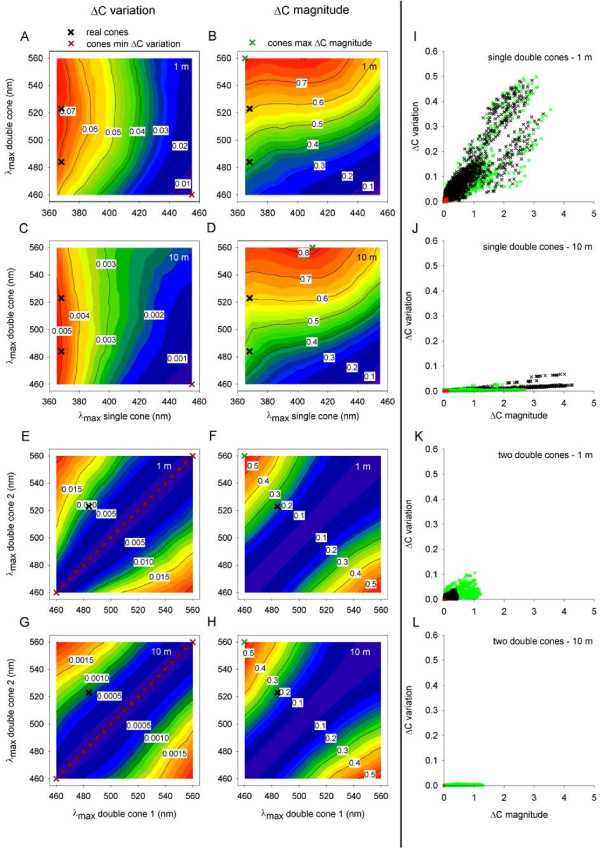
**Spectral location of pigments in *Metriaclima zebra *is tuned to allow large chromatic contrast between fish pattern and background. (A**-**H)** Median Δ*C* between fish pattern and background. Δ*C* was modeled for opponent channels that compare the outputs of one single cone and one double cone **(A**-**D)**, and channels that compare the outputs of two double cones **(E**-**H)**. The various colors and tags in the contour plots represent different median Δ*C* values. The maxΔ*C*mag pigment pairs comprised pigments with λ_max_ that were far apart, and resembled the λ_max_ of the real pigments in *M. zebra* (368, 484, and 523 nm). In contrast, the minΔ*C*var pigment pairs consisted of pigments with λ_max_ that were closer together. This trend held for both opponent channel types and for both 1 m and 10 m depth. **(I**-**L)** Scatterplots illustrating the relationship between Δ*C* magnitude and Δ*C* variation for the real (black), minΔ*C*var (red), and maxΔ*C*mag (green) pigment pairs. Δ*C* magnitude produced by the real pigment pair resembled the Δ*C* amplitude produced by the maxΔ*C*mag pigment pair better than it resembled the Δ*C* amplitude produced by the minΔ*C*var. This was observed at both 1 m and 10 m depth, and for both opponent channel types **(I**,**J** and **K**,**L)**. Δ*C* variation at 10 m depth was smaller than at 1 m depth. Regardless of water depth, however, the real and maxΔ*C*mag pigment pairs produced variation in Δ*C* that was larger than in the minΔ*C*var pigment pairs. Sample size, *n* = 696 for either the real, minΔ*C*var, or maxΔ*C*mag pigment pairs, for each of the opponent channels and water depths examined.

The variation and magnitude of Δ*C* for an opponent channel formed by one single cone and one double cone located at a depth of 1 m are plotted for various cone pigment pairs in Figure 4A and B, respectively. Calculated values are displayed in the white tags on the isovalue contours across the various pigment combinations. Values rise from blue to red regions on the plots. Figure 4C,D presents findings for the same model channel located at a depth of 10 m. Results for similar analysis of an opponent pair of double cones are plotted in Figure 4E-H. For the opponent channel formed by comparison of one single cone and one double cone, the maxΔ*C*mag pigment pair constituted of pigments with λ_max_ that were far apart (365 and 560 nm for 1 m depth; 410 and 560 for 10 m depth; Figure 4B,D, green X symbols), while the minΔ*C*var pigment pair constituted of pigments with λ_max_ that were closer together (455 and 460 for both 1 and 10 m depth; Figure 4A,C, red X symbols). Similarly, for the opponent channel formed by comparison of two double cones, the maxΔ*C*mag pigment pair constituted of pigments with λ_max_ that were far apart (460 and 560 nm for both 1 and 10 m depth), while the minΔ*C*var pigment pairs constituted of two identical pigments with λ_max_ spanning the realistic range for double cones (460 to 560 nm), for both 1 and 10 m depth (Figure 4E-H). Thus, the λ_max_ of the real pigments in *M. zebra* (368, 484, and 523 nm; black X symbols)*,* and the spectral separation between the pigments, resemble those of the maxΔ*C*mag pigments more than they resemble those of the minΔ*C*var pigments.

Next, we examined quantitatively how the Δ*C* magnitude produced by the actual cone pigments in *M. zebra* corresponds to the Δ*C* magnitudes produced by the maxΔ*C*mag and minΔ*C*var pigments. Because of the large sample size (*n* = 696 for each of the groups, 87 body patterns × 8 rock substrates), slight differences between treatment groups most often resulted in statistically significant differences between groups (*P* <0.05). We therefore chose to report observed effect size (that is, *η*^2^ values) to allow evaluation of the magnitude of difference between groups. The larger the effect size, the larger is the difference between treatment groups. Unless specified differently, *P* for all comparisons was <0.05.

For the opponent channel formed by one single cone and one double cone (Figure 4I,J), Δ*C* magnitude differed between the real (black), minΔ*C*var (red), and maxΔ*C*mag (green) pigment pairs (RT, df = 3, *N* = 696 for each group, *η*^2^ = 0.243 and 0.186 for 1 and 10 m depth). *Post-hoc* analysis revealed that Δ*C* magnitude for the minΔ*C*var pigment pair was smaller than for either the real pigment pairs (SWS1-Rh2b, df = 1, *η*^2^ = 0.312 and 0.253 for 1 and 10 m depth; SWS1-Rh2a, df = 1, *η*^2^ = 0.362 and 0.289 for 1 and 10 m depth) or the maxΔ*C*mag pigment pair (df = 1, *η*^2^ = 0.422 and 0.446 for 1 and 10 m depth). In contrast, Δ*C* magnitude for the real and maxΔ*C*mag pigment pairs differed only slightly (RT, df = 2, *η*^2^ = 0.027 and 0.005 for 1 and 10 m depth), as did the Δ*C* magnitude for the real pigment pairs (RT, df = 1, *η*^2^ = 0.013 and 0.006 for 1 and 10 m depth). Therefore, the Δ*C* magnitude produced by the real and maxΔ*C*mag pigment pairs is largely similar, but considerably larger than the Δ*C* magnitude produced by the minΔ*C*var pigment pair.

For the opponent channel formed by two double cones (Figure 4K,L), Δ*C* magnitude differed between the real, minΔ*C*var, and maxΔ*C*mag pigment pairs (RT, df = 2, *N* = 696 for each group, *η*^2^ = 0.555 and 0.556 for 1 and 10 m depth). *Post-hoc* analysis revealed that Δ*C* magnitude for the minΔ*C*var pigment pair was smaller than for either the real pigment pair (Rh2b-Rh2a, df = 1, *η*^2^ = 0.555 and 0.556 for 1 and 10 m depth) or the maxΔ*C*mag pigment pair (df = 1, *η*^2^ = 0.585 and 0.585 for 1 and 10 m depth). In contrast, Δ*C* magnitude for the real and maxΔ*C*mag pigment pairs differed less considerably (RT, df = 1, *η*^2^ = 0.315 and 0.316 for 1 and 10 m depth). Thus, the Δ*C* magnitude produced by the real pigment pair resembles the Δ*C* magnitude produced by the maxΔ*C*mag pigment pair better than it resembles the Δ*C* magnitude produced by the minΔ*C*var. Therefore, at both 1 and 10 m depth, the real and maxΔ*C*mag pigment pairs are more effective than the minΔ*C*var pigment pair in producing large Δ*C* magnitude. These results suggest that the spectral location of pigments in *M. zebra* is almost optimally tuned to allow the largest possible Δ*C* magnitude between fish pattern and background, as predicted by the contrast theory.

Figure 4I-L allows comparison of the Δ*C* variation produced by the real cone pigments in *M. zebra* with that produced by the maxΔ*C*mag and minΔ*C*var pigments. For the opponent channel formed by one single cone and one double cone (Figure 4I,J), the flicker-induced variation in Δ*C* differed between the real, minΔ*C*var, and maxΔ*C*mag pigment pairs (RT, df = 3, *N* = 696 for each group, *η*^2^ = 0.189 and 0.197 for 1 and 10 m depth). *Post-hoc* analysis revealed that Δ*C* variation for the minΔ*C*var pigment pair was smaller than for either the real pigment pairs (SWS1-Rh2b, df = 1, *η*^2^ = 0.314 and 0.269 for 1 and 10 m depth; SWS1-Rh2a, df = 1, *η*^2^ = 0.319 and 0.263 for 1 and 10 m depth) or the maxΔ*C*mag pigment pair (df = 1, *η*^2^ = 0.318 and 0.372 for 1 and 10 m depth). However, Δ*C* variation for the real and maxΔ*C*mag pigment pairs did not differ significantly at 1 m depth (RT, df = 2, *P* = 0.257, *η*^2^ = 0.001), but did differ at 10 m depth (RT, df = 2, *P* <0.001, *η*^2^ = 0.071); At both 1 and 10 m depth, Δ*C* variation for the real pigment pairs did not differ significantly (RT, df = 1, *P* = 0.798 and *η*^2^ = 0.0008 for 1 m depth, *P* = 0.666 and *η*^2^ <0.0001 for 10 m depth).

For the opponent channel formed by two double cones (Figure 4K,L), the flicker-induced variation in Δ*C* differed between the real, minΔ*C*var, and maxΔ*C*mag pigment pairs (RT, df = 2, *N* = 696 for each group, *η*^2^ = 0.458 and 0.563 for 1 and 10 m depth). *Post-hoc* analysis revealed that Δ*C* variation for the minΔ*C*var pigment pair was smaller than for either the real pigment pair (Rh2b-Rh2a, df = 1, *η*^2^ = 0.458 and 0.563 for 1 and 10 m depth) or the maxΔ*C*mag pigment pair (df = 1, *η*^2^ = 0.488 and 0.598 for 1 and 10 m depth). In contrast, Δ*C* variation for the real and maxΔ*C*mag pigment pairs differed less substantially (RT, df = 1, *η*^2^ = 0.023 and 0.318 for 1 and 10 m depth). Therefore, the real pigment pairs are often slightly more effective than the maxΔ*C*mag pigment pairs in eliminating the variation in Δ*C*. Nevertheless, at both 1 and 10 m depth, the real and maxΔ*C*mag pigment pairs are considerably less effective than the minΔ*C*var pigment pairs in eliminating the variation in Δ*C*. These results suggest that the spectral location of pigments in *M. zebra* is poorly tuned to allow elimination of temporal fluctuations in the visual signal, in contrast to the prediction of the flicker theory.

Taken together, three lines of evidence suggest that the spectral location of cone pigments in *M. zebra* has been shaped as predicted by the contrast theory rather by the flicker theory. These are: (i) similarity in cone spectral locations between the maxΔ*C*mag pigments and the real pigments in *M. zebra*, (ii) greater efficiency of the real and maxΔ*C*mag pigment pairs in producing large Δ*C* magnitude as compared to the minΔ*C*var pigment pair, and (iii) lower efficiency of the real and maxΔ*C*mag pigment pairs in eliminating the variation in Δ*C*, as compared to the minΔ*C*var pigment pair. Thus, the spectral location of pigments in *M. zebra* is tuned to produce large Δ*C* magnitude between fish pattern and background, and is poorly tuned to allow elimination of temporal fluctuations in the visual signal. That is, the visual system of *M. zebra* is tuned as predicted by the contrast theory rather than by the flicker theory (or by both theories).

## Conclusions

Our results show that the amplitude of the light flicker and the distribution of its power across temporal frequencies vary across the light spectrum, violating the flicker theory’s first assumption. We examined the effect of the wavelength dependence of light flicker on the output of cones and found that, contrary to the prediction of the flicker theory, simple subtraction of the output of one cone class from that of another through opponent interactions would not produce a flicker-free output signal. Moreover, neither fixed low pass filtration nor adjustment of dynamic range of cones would likely to be favored. Thus, although there might be a mechanism by which flicker-free visual signals would be generated under flickering illumination, the likelihood of such a possibility is low. Importantly, even if such generation of a flicker-free visual signal would prove possible, our results show that the temporal frequency of flicker matches the frequency where sensitivity is maximal in a wide range of fish species, suggesting that the flicker may potentially enhance the detection of objects. Thus, there appears to be no real need to eliminate the flicker, because, in contrast to the accepted belief and the second assumption of the flicker theory, the flicker can most likely improve the detection of objects rather than degrade it. The violation of its two critical assumptions suggests little support for the flicker theory as originally formulated. While this alone does not support the contrast theory, comparison of the contrast and flicker theories by means of chromatic contrast modeling under flickering illumination revealed that the visual system of our focal species was tuned as predicted by the contrast theory rather than by the flicker theory. This suggests that the main factor that has tuned the spectral locations of cone pigments is the optimization of visual contrast. Thus, we propose that the contrast theory, stating that multiple cone classes evolved to maximize the visual contrast between objects and backgrounds, is the most parsimonious at present. This result may have important implications for our understanding of the adaptive significance of the number and spectral tuning of cone pigments and the characteristics of retinal networks in vertebrate visual systems.

## Methods

### Measurement of underwater light flicker

The study was conducted on 21 July 2008 at a near-shore site at Cape Maclear, Nankumba Peninsula, Lake Malawi. The sampling site (14° 01’ 26.42” S 34° 49’ 25.91” E) was located on the southern shore of Thumbi West Island. This site is exposed to wind and wave action [55] and has a rock-sand transition depth of approximately 12 m.

To study the light flicker characteristics, downward and sideward irradiance was measured at a high sampling rate. Irradiance was measured using a thermoelectrically cooled spectroradiometer (QE65000, Ocean Optics, Dunedin, FL, USA) connected to a 30 m optical fiber (ZPK600-30 Ultraviolet–visible, Ocean Optics) that was fitted with a cosine corrector (diameter = 3.9 mm; CC-3-Ultraviolet, Ocean Optics). This diameter of the cosine corrector was expected to accurately capture the irradiance fluctuations at near-surface depths. However, we cannot exclude the possibility of miscapturing fluctuations of small spatial scale, typically encountered at depths smaller than 1 m [22]. The spectroradiometer employed a 1,024 × 58-element square silicon charge-coupled device (CCD) array, configured with a 25 μm slit and a variable blaze wavelength grating (HC-1, groove density = 300 mm^-1^, Ocean Optics), resulting in an effective spectral resolution of 1.9 nm ‘Full Width at Half Maximum’ (FWHM) between 200 and 950 nm. The spectroradiometer’s integration time was set to 25 ms (theoretical sampling frequency = 40 Hz) to allow for the highest possible sampling rate while ensuring sufficiently high signal-to-noise ratio. In practice, however, due to a time constant between successive readings, the actual sampling frequency was 17.34 Hz. Thus, 3,000 measurements were saved over 173 s, constituting a measurement time series. The spectroradiometer setup was calibrated for absolute irradiance prior to measurement using a calibrated halogen-deuterium dual light source (200 to 1,000 nm, DH-2000-CAL, Ocean Optics). The optical fiber head was mounted on a 1 m tall tripod, 1, 2, 4, 6, and 10 m below the water surface, and readings were saved on a laptop computer placed on a boat. To prevent shading, the boat was positioned as far as possible from the tripod and never between the tripod and the sun. Irradiance measurements were conducted under clear blue sky, at 12:20 to 14:09 (local time), with solar zenith angles of 46° to 55°, and under light winds of 1.8 m/s.

Note that various elements included in the spectroradiometer setup may introduce spectrally-specific variation in the measurement. These include: (i) light attenuation in the fiber optic cable, (ii) light absorbance by the spectroradiometer’s mirrors, (iii) reflectance efficiency of the spectroradiometer’s grating, and (iv) response of the CCD detector. However, the wavelength dependence of these elements has been removed by calibrating the spectroradiometer setup (including the spectroradiometer, fiber optic and cosine corrector) for absolute irradiance. Therefore, spectrally-specific differences in the design of the spectroradiometer setup likely had little effect (if any) on the observed wavelength dependence of light flicker.

Although vision is essentially a task of low spectral resolution and high temporal resolution radiance detection, we have chosen to measure irradiance at a relatively low temporal resolution for several reasons. First, we aimed at investigating the wavelength dependence of light flicker, so we chose to sacrifice some temporal resolution while ensuring precise representation of irradiance across the spectrum. Second, we chose to focus on characterization of light flicker at temporal frequencies corresponding to the F_max_ of fish;, typically ranging between 2 and 4 Hz (Figure 3). Third, the power of light flicker typically declines steeply with increasing temporal frequency. For example, the power of light flicker at a frequency of 8.67 Hz (our frequency limit considering a sampling frequency of 17.34 Hz) was reported to be approximately 5 to 200 fold smaller than that at the dominant frequency at depths of 0.86 to 2.84 m [56]. Indeed, to fully capture the highest-frequency irradiance fluctuations, it would be necessary to use a high rate (for example, 1 kHz) radiometric measurement system [22]. However, the relatively low frequency of F_max_ in fish as well as the steep decline of light flicker’s power with increasing frequency, suggest a limited effect of high-frequency irradiance fluctuations on the appearance of objects.

### Analysis of amplitude and temporal frequency of light flicker

To standardize the 3,000 readings included in each irradiance time series, the noise level (measured with the tip of the cosine corrector blocked) was subtracted from each spectroradiometer reading, and the resulting reading in relative counts was converted into photon irradiance. Wavelengths at which irradiance was lower than 3 × 10^11^ photons cm^2^/s/nm were designated as unreliable and removed from further analysis. To estimate the amplitude of the light flicker, we calculated the CV of each irradiance time series. CV is commonly used in describing the variation in irradiance and radiance flicker [22,34,35,57]. To study the frequency characteristics of the wave-induced light flicker, we calculated the power spectrum of temporal frequencies for each irradiance time series at a light wavelength resolution of 1 nm. Specifically, the discrete Fourier transform (DFT) was calculated for each time series by using the fast Fourier transform (FFT) algorithm, and while applying a Hamming frequency window that is appropriate for analyzing closely spaced sine waves [58]. Additionally, as indices of the distribution of power across frequencies, we calculated the fP_50_, fP_90_, fP_99_ that stand for the frequencies corresponding to 50, 90, and 99 percent of the cumulative power of the light flicker. Although dependent on the frequency range examined, the fP indices describe the modulations experienced by an observer reasonably well [40]. Finally, to assess the wavelength dependence of the power distribution of light flicker, we calculated the root mean square error (RMSE) and the normalized RMSE (NRMSE) between the power distribution at 500 and 550 nm. Irradiance at these wavelengths is highest and most reliable, and thus, the calculated RMSE and NRMSE are likely to serve as good estimates for wavelength dependence. NRMSE equaled RMSE divided by the difference between the maximum and minimum power across the spectrum.

### Modeling the magnitude and variation of chromatic contrast under flickering illumination

Chromatic contrast modeling was performed following Kelber *et al*. [59] and Cummings [60]. The quantum catch of each cone photoreceptor, *Q*_i,_ when viewing a given color patch of the fish was calculated according to:

(1) $$Q_{i}=\int_{300}^{800}R_{t}\left(\lambda \right)E_{h}\left(\lambda \right)A_{i}\left(\lambda \right)T\left(\lambda \right)\mathrm{d\lambda}$$

where *R*_t_(λ) is the spectral reflectance of the target (ranging 0 to 1), *E*_h_(λ) is the normalized sideward spectral irradiance incident on the object (ranging 0 to 1), *A*_i_(λ) is the normalized absorbance of cone photoreceptor *i* (ranging 0 to 1), and *T*(λ) is the normalized spectral transmission of the ocular media (ranging 0 to 1). Similarly, the quantum catch of each photoreceptor when viewing the background of a rock substrate (the stimulus fish might be viewed against) was calculated using Equation 1 where *R*_t_(λ) was substituted by the spectral reflectance of the substrate, *R*_b_(λ). The absorbance of photoreceptors was estimated as the empirical absorbance templates of visual pigments given by Govardovskii *et al.*[61]. See below detailed procedures for the measurement of spectral reflectance of the body pattern of fish and rock substrate (approximated by diffuse reflectance [62]), and spectral transmission of the ocular media (approximated by the transmission of the lens [63]). The quantum catch of photoreceptors should ideally be estimated using absorbtance rather than absorbance spectra, with the former depending on the transverse specific density of pigments and the outer segment length of photoreceptors. However, transverse specific density and outer segment length data in *M. zebra* (our focal species) and in African cichlids as a whole is largely unexplored, with the few available reports providing incomplete and contradicting values [64-66]. Thus, photoreceptor quantum catch was estimated using absorbance spectra.

To account for the light adaptation properties of photoreceptors, photoreceptor quantum catches, *Q*_i_, were normalized to the adapting background irradiance by the von Kries coefficients, *K*_i_:

(2) $$q_{i}=K_{i}\;Q_{i}$$

These *K*_i_ coefficients were chosen so that the quantum catches for the adapting irradiance is constant, that is:

(3) $$K_{i}=1/\int_{300}^{800}{\bar{E}}_{h}\left(\lambda \right)A_{i}\left(\lambda \right)T\left(\lambda \right)\mathrm{d\lambda}$$

where ${\bar{E}}_{h}\left(\lambda \right)$ is the normalized mean sideward spectral irradiance that was assumed to adapt the fish eye, calculated as the time-average of 3,000 consecutive sideward irradiance measurements. We modeled two types of opponent channels: (i) a channel that compares the outputs of one single cone and one double cone [*C*_sd_], and (ii) a channel that compares the outputs of two double cones [*C*_dd_]:

(4) $$\begin{array}{l} C_{\mathrm{sd}}=q_{s}-q_{d}\\ C_{\mathrm{dd}}=q_{d1}-q_{d2} \end{array}$$

Thereafter, we calculated the chromatic contrast (Δ*C*), formed by comparison of the output of a given opponent channel when viewing the body color pattern of fish and the background against which it might be viewed:

(5) $$\Delta C=C_{t}-C_{b}$$

where *C*_t_ and *C*_b_ represent the output of a given opponent channel when viewing the object and the background, respectively. Δ*C* amplitude was estimated as the time average of Δ*C* over 100 consecutive high-temporal resolution sideward irradiance measurements (total duration = 6 s), and Δ*C* variation was estimated as the standard deviation in Δ*C* over time. The use of standard deviation, rather than coefficient of variation, to estimate the variation in Δ*C* is appropriate because the quantum catches of photoreceptors were already normalized to the mean adapting irradiance; this effectively rendered the quantum catches of the different cones to be of the same magnitude.

### Measurement of spectral reflectance of the body pattern of fish

Diffuse spectral reflectance of the body pattern of *M. zebra* (*n* = 87) was measured at 1-nm intervals using a spectroradiometer (effective spectral resolution = 2.06 nm FWHM for 200 to 950 nm; USB2000, Ocean Optics) connected to one arm of a 2 m bifurcated optical fiber (BIF600-2 Ultraviolet–visible, Ocean Optics). The other arm of the fiber was connected to a high output light source (200 to 1,000 nm; DH-2000-BAL, Ocean Optics). The common end of the bifurcated fiber was fitted with a flat black reflectance probe that showed a 3 mm diameter tip, cut at an angle of 45°. A measurement of a Spectralon diffuse reflectance standard (WS-1-SL, Ocean Optics) was taken as 100% reflectance, and a dark measurement was taken as zero reflectance. Fish were immersed in 500 ml water containing 2 ml of 1:10 clove oil:ethanol solution immediately after capture until the fish reached stage III anesthesia [67]. Reflectance was measured at 16 to 23 different points across the submerged fish body of 5 individuals. All experimental and animal care procedures were approved by Queen’s University Animal Care Committee under the auspices of the Canadian Council for Animal Care.

### Measurement of spectral reflectance of rock substrate

Diffuse spectral reflectance of rock substrate (*n* = 8) was measured at a near-shore site in Lake Malawi (14° 00’ 58.02” S 34° 48’ 33.29” E) [68]. Rock reflectance was measured using a custom-built probe that included a diving flashlight (mini Q40, Underwater Kinetics, Poway, CA, USA) and a fiber-coupled spectroradiometer (Jaz, Ocean Optics). The tip of the flashlight was fitted with an adaptor that held the optical fiber (QP600-2 Ultraviolet–visible, Ocean Optics) oriented at an angle of 45° to the examined surface. The far side of the adaptor included a ring of black foam that sealed the reflectance probe against the surface examined. A SCUBA diver held the reflectance probe against rock substrates while readings were acquired and saved on a laptop computer in a boat. The irradiance spectrum of the flashlight allowed reliable reflectance measurements between 370 and 800 nm, and the spectroradiometer configuration resulted in an effective spectral resolution of 2.06 nm (FWHM) across this range. A measurement of a Spectralon diffuse reflectance standard was taken as 100% reflectance, and a dark measurement was taken as zero reflectance.

### Measurement of spectral transmission of fish lens

Spectral transmission of the fish lens was measured following a protocol described elsewhere [69,70]. Lenses were surgically removed from the eyes and were mounted in a hole that was drilled in a black plastic block fitted inside a standard sample cuvette. Transmission measurements between 300 and 750 nm were carried out using a bench-top spectrophotometer (Cary 300; Varian, Palo Alto, CA, USA) and were normalized between 0 and 1. For each fish (*n* = 3), six to ten transmission measurements were acquired from both lenses and averaged.

### Statistical analysis

F_max_ of fish from different water depth categories and habitat types did not follow normal distribution (Kolmogorov-Smirnov test) and differed in variance (Leven’s test). Thus, to test the effect of water depth and habitat type on F_max_, we used ANOVA permutation tests, with the difference between the means of the various depth categories and habitat types as a test statistic (R package ‘lmPerm’, maximum number of iterations = 50,000, *α* = 0.05) [71]. Similarly, chromatic contrast (Δ*C*) between fish pattern and background for the real, minΔ*C*var, and maxΔ*C*mag pigment pairs did not follow normal distribution and their variance differed between groups. Thus, permutation tests were used also to test the effect of pigment pair on Δ*C* amplitude and variation. To allow evaluation of the magnitude of difference in Δ*C* amplitude and variation between pigment pair treatments, effect size was estimated as *η*^2^ (= sum of squares treatment/sum of squares total). Statistical analysis was performed using R 3.0.0 (The R Foundation for Statistical Computing).

## Competing interests

The authors declare that they have no competing interests.

## Authors’ contributions

SS conceived and designed the study, analyzed the data, and wrote the manuscript; SS and CWH performed all the experiments. Both authors have read and approved the final manuscript.

## Supplementary Material

Additional file 1 **Temporal frequency of light flicker is wavelength dependent across various water depths. (A-D)** The frequency distribution of the flicker in downward irradiance at a depth of 2 m (A), 4 m (B), 6 m (C), and 10 m (D) differed across the light spectrum. For clear graphical presentation, the power spectrum of light flicker, normalized to the dominant frequency (1.54 Hz for 2 m, 0.83 Hz for 4 m, 0.80 Hz for 6 m, 0.67 Hz for 10 m) is presented for different wavelengths at 50 nm intervals. **(E-G)** Cumulative power of wave-induced flicker across wavelengths and water depths. As indices of the distribution of flicker power across temporal frequencies, we calculated the fP_50_, fP_90_, and fP_99_ that stand for the temporal frequencies that correspond to 50, 90, and 99 percent of the cumulative power of wave-induced flicker. fP_50_, fP_90_, and fP_99_ increased toward longer light wavelengths, further supporting the wavelength dependence of the temporal frequency structure of flicker. Note that deeper in the water column, the irradiance at both ends of the spectrum was too low to be considered reliable (see Methods for criteria for excluding data points); therefore, the spectral range presented narrows with depth. Click here for file

Additional file 2 **Amplitude and temporal frequency of the light flicker in sideward irradiance are wavelength dependent. (A,B)** Examples of light flicker time series of sideward irradiance at 1 m depth and light wavelengths of 400 and 600 nm. The amplitude of the light flicker at 600 nm is larger than at 400 nm. **(C)** The amplitude of light flicker in sideward irradiance decreased with growing water depth, and increased monotonically toward longer light wavelengths. The ratio between the amplitude at the longest and shortest wavelengths did not vary considerably across depths, and ranged between 2.26 and 2.79 (presented next to each spectrum). **(D)** The frequency distribution of the flicker at a depth of 1 m differed across the light spectrum. The power spectrum of light flicker, normalized to the dominant frequency (1.69 Hz), is presented for different wavelengths at 50 nm intervals. The frequency distribution of flicker at 2, 4, 6, and 10 m depth also differed between wavelengths (not presented). **(E)** The frequency distribution of light flicker at 500 nm differed across water depths, with the dominant frequency (1 m, 1.69 Hz; 2 m, 1.30 Hz; 4 m, 0.83 Hz; 6 m, 0.78 Hz; 10 m, 0.59 Hz) and the relative power at high frequencies decreasing with growing depth. **(F)** The wavelength dependence of light flicker became weaker with growing depth. Click here for file

Additional file 3 **Compilation of critical fusion frequency (CFF) and the frequency at which maximum contrast sensitivity is attained (F**_**max**_**, estimated as 15% of CFF) in fish.** Frequencies are given in Hz. Click here for file

Additional file 4 **Comparison between the frequency of light flicker in downward irradiance and two realistic estimates of F**_**max**_**.** F_max_ was estimated as either 10% **(A,B)** or 20% **(C,D)** of CFF. (A,C) The distribution of F_max_ values across frequency corresponded well to the power spectrum of flicker across depths. Note, however, that estimation of F_max_ as 20% of CFF resulted in F_max_ values that often exceeded the sampling frequency limit of light flicker. Depicted power spectrum (shaded gray) represents the envelope of flicker power across the 1 m and 10 m depth range. (B,D) Comparison between the cumulative power of the flicker and F_max_ for dim and bright stimuli (closed circles). Conventions for the indices of the distribution of power of flicker across frequencies (fP_50_, fP_90_, and fP_99_), plot specifications, and species included in the analysis are the same as in Figure 3A,B. For estimation of F_max_ as 10% of CFF, the median F_max_ equaled 1.2 and 2.3 Hz, for dim and bright stimuli, respectively (open circle; red error bars represent the 25th and 75th percentiles). The median F_max_ for dim and bright stimuli matched the frequency below which approximately 50% of the cumulative power of flicker was found at 1 m depth. For estimation of F_max_ as 20% of CFF, the median F_max_ equaled 2.4 and 4.6 Hz, for dim and bright stimuli, respectively. The median F_max_ for dim and bright stimuli matched the frequency below which between 50% and 90% of the cumulative power of flicker was found at 1 m depth. For both F_max_ estimates, the median F_max_ for dim and bright stimuli matched the frequency below which 99% of the cumulative power at 10 m depth was found. Click here for file

Additional file 5 **Spectra used for chromatic contrast modelling**. **(A,B)** A total of 100 spectra of sideward irradiance at a water depth of 1 m (A) and 10 m (B). These spectra were taken as the irradiance that illuminated the stimulus fish and the vertical rock substrate. **(C,D)** Mean spectral sideward irradiance at a depth of 1 m (C) and 10 m (D) that was taken as the irradiance that adapted the viewer fish eye. **(E)** Spectral reflectance of the body pattern of fish (*n* = 87). **(F)** Spectral reflectance of diverse rock substrates (*n* = 8). **(G)** Spectral transmission of the lens in *Metriaclima zebra*. **(H)** Spectral absorbance templates for visual pigments of A_1_ chromophore constructed based on the cone pigments typically found in adult *M. zebra*: a single cone-occupying pigment (SWS1, λ_max_ = 368 nm) and two double cones-occupying pigments (Rh2b, λ_max_ = 484 nm; Rh2a, λ_max_ = 523 nm). For graphical presentation only, each of the spectra presented in (A-F) was normalized by its norm. Click here for file
