# Supplementary figures and images for: What has driven the evolution of multiple cone classes in visual systems: object contrast enhancement or light flicker elimination?
Source: BMC Biol. 2013 Jul 4;11:77. doi: 10.1186/1741-7007-11-77 (PMC3720213; doi:10.1186/1741-7007-11-77)

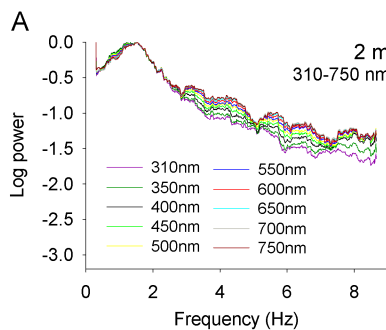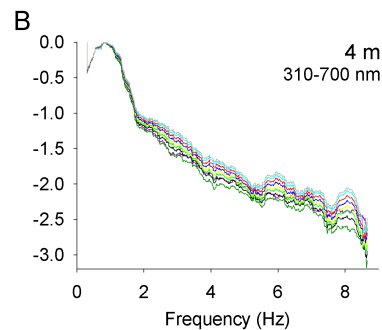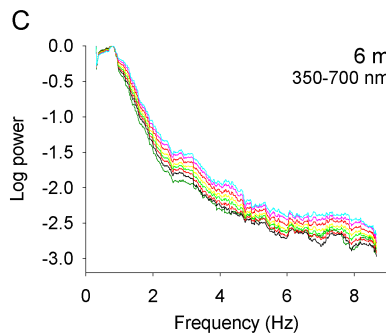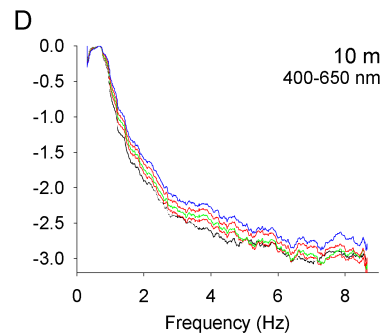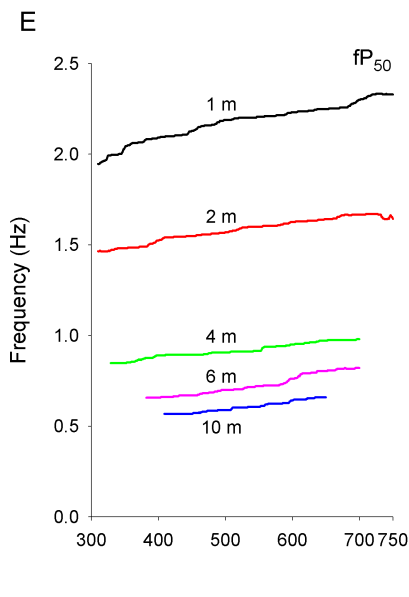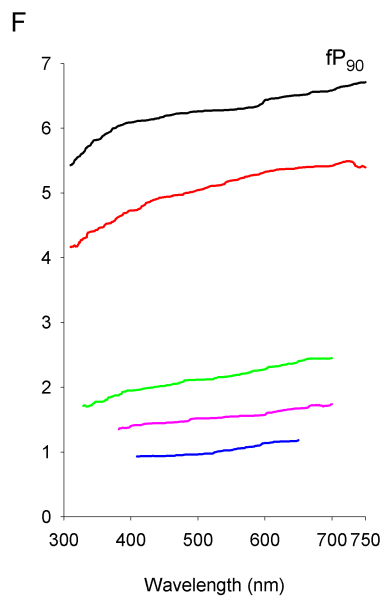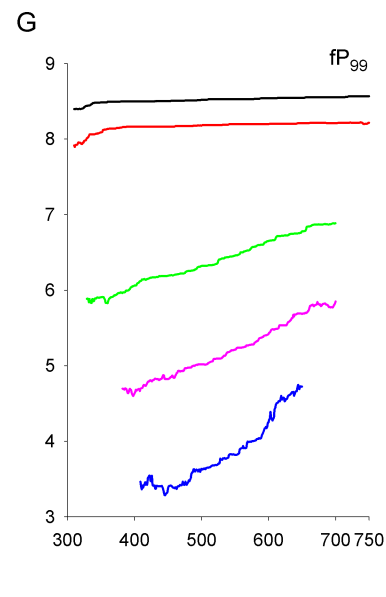

Supplement: Additional file 1 — Temporal frequency of light flicker is wavelength dependent across various water depths. (A-D) The frequency distribution of the flicker in downward irradiance at a depth of 2 m (A), 4 m (B), 6 m (C), and 10 m (D) differed across the light spectrum. For clear graphical presentation, the power spectrum of light flicker, normalized to the dominant frequency (1.54 Hz for 2 m, 0.83 Hz for 4 m, 0.80 Hz for 6 m, 0.67 Hz for 10 m) is presented for different wavelengths at 50 nm intervals. (E-G) Cumulative power of wave-induced flicker across wavelengths and water depths. As indices of the distribution of flicker power across temporal frequencies, we calculated the fP50, fP90, and fP99 that stand for the temporal frequencies that correspond to 50, 90, and 99 percent of the cumulative power of wave-induced flicker. fP50, fP90, and fP99 increased toward longer light wavelengths, further supporting the wavelength dependence of the temporal frequency structure of flicker. Note that deeper in the water column, the irradiance at both ends of the spectrum was too low to be considered reliable (see Methods for criteria for excluding data points); therefore, the spectral range presented narrows with depth. [file 1741-7007-11-77-S1.pdf]

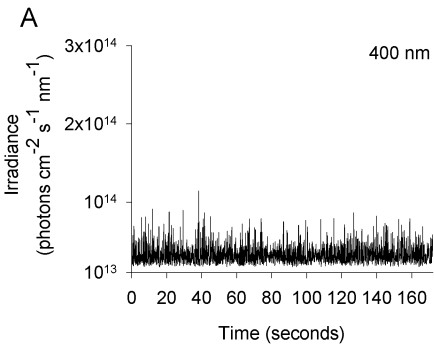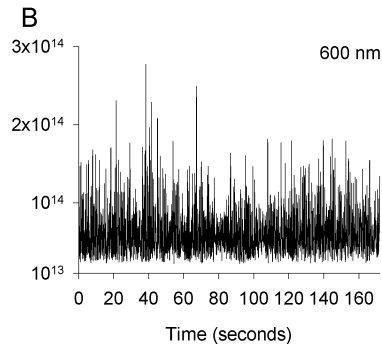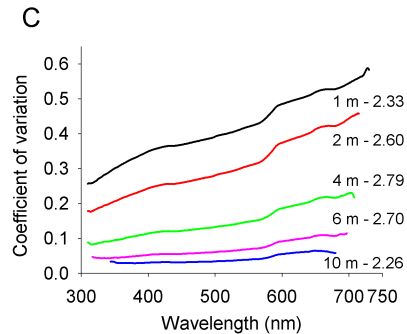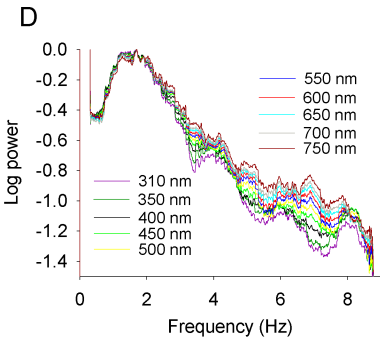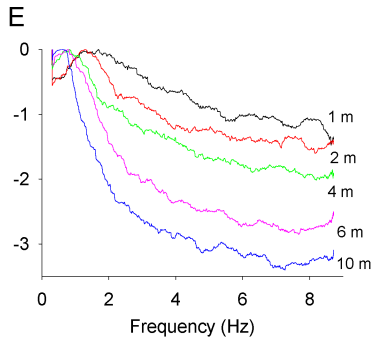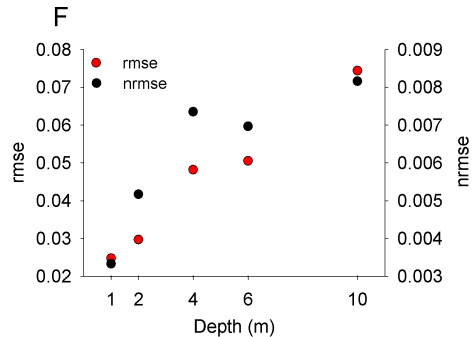

Supplement: Additional file 2 — Amplitude and temporal frequency of the light flicker in sideward irradiance are wavelength dependent. (A,B) Examples of light flicker time series of sideward irradiance at 1 m depth and light wavelengths of 400 and 600 nm. The amplitude of the light flicker at 600 nm is larger than at 400 nm. (C) The amplitude of light flicker in sideward irradiance decreased with growing water depth, and increased monotonically toward longer light wavelengths. The ratio between the amplitude at the longest and shortest wavelengths did not vary considerably across depths, and ranged between 2.26 and 2.79 (presented next to each spectrum). (D) The frequency distribution of the flicker at a depth of 1 m differed across the light spectrum. The power spectrum of light flicker, normalized to the dominant frequency (1.69 Hz), is presented for different wavelengths at 50 nm intervals. The frequency distribution of flicker at 2, 4, 6, and 10 m depth also differed between wavelengths (not presented). (E) The frequency distribution of light flicker at 500 nm differed across water depths, with the dominant frequency (1 m, 1.69 Hz; 2 m, 1.30 Hz; 4 m, 0.83 Hz; 6 m, 0.78 Hz; 10 m, 0.59 Hz) and the relative power at high frequencies decreasing with growing depth. (F) The wavelength dependence of light flicker became weaker with growing depth. [file 1741-7007-11-77-S2.pdf]

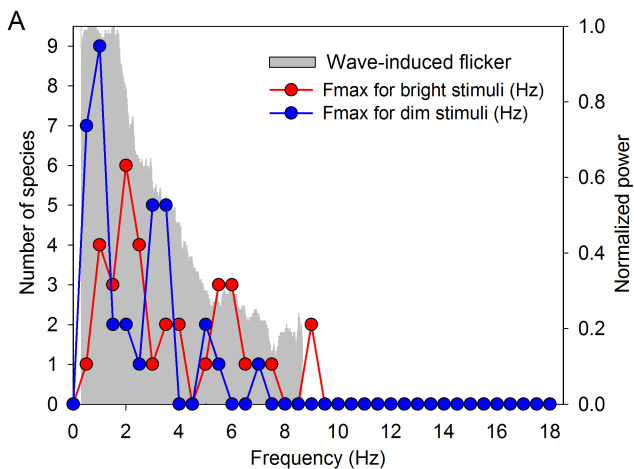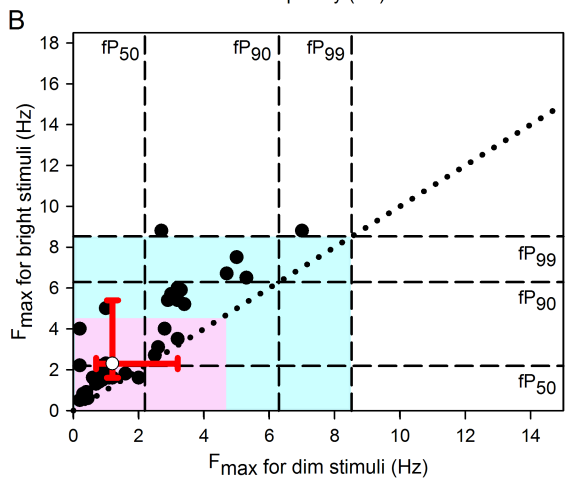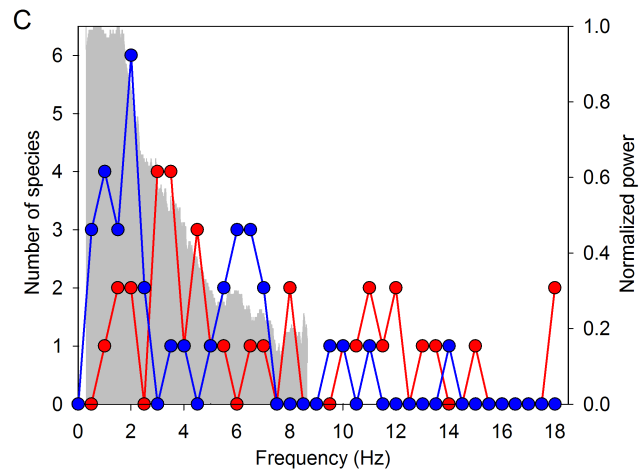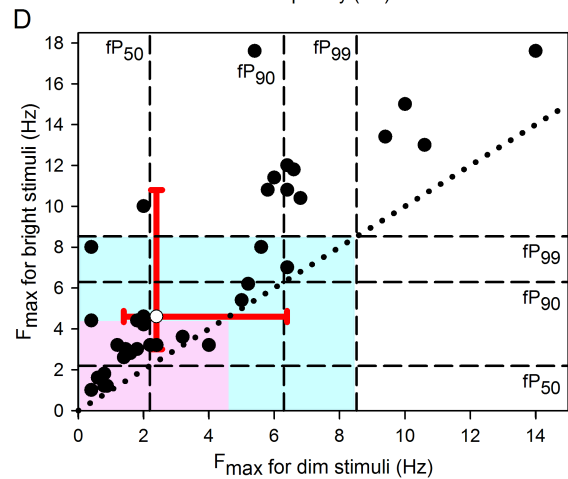

Supplement: Additional file 4 — Comparison between the frequency of light flicker in downward irradiance and two realistic estimates of Fmax. Fmax was estimated as either 10% (A,B) or 20% (C,D) of CFF. (A,C) The distribution of Fmax values across frequency corresponded well to the power spectrum of flicker across depths. Note, however, that estimation of Fmax as 20% of CFF resulted in Fmax values that often exceeded the sampling frequency limit of light flicker. Depicted power spectrum (shaded gray) represents the envelope of flicker power across the 1 m and 10 m depth range. (B,D) Comparison between the cumulative power of the flicker and Fmax for dim and bright stimuli (closed circles). Conventions for the indices of the distribution of power of flicker across frequencies (fP50, fP90, and fP99), plot specifications, and species included in the analysis are the same as in Figure 3A,B. For estimation of Fmax as 10% of CFF, the median Fmax equaled 1.2 and 2.3 Hz, for dim and bright stimuli, respectively (open circle; red error bars represent the 25th and 75th percentiles). The median Fmax for dim and bright stimuli matched the frequency below which approximately 50% of the cumulative power of flicker was found at 1 m depth. For estimation of Fmax as 20% of CFF, the median Fmax equaled 2.4 and 4.6 Hz, for dim and bright stimuli, respectively. The median Fmax for dim and bright stimuli matched the frequency below which between 50% and 90% of the cumulative power of flicker was found at 1 m depth. For both Fmax estimates, the median Fmax for dim and bright stimuli matched the frequency below which 99% of the cumulative power at 10 m depth was found. [file 1741-7007-11-77-S4.pdf]

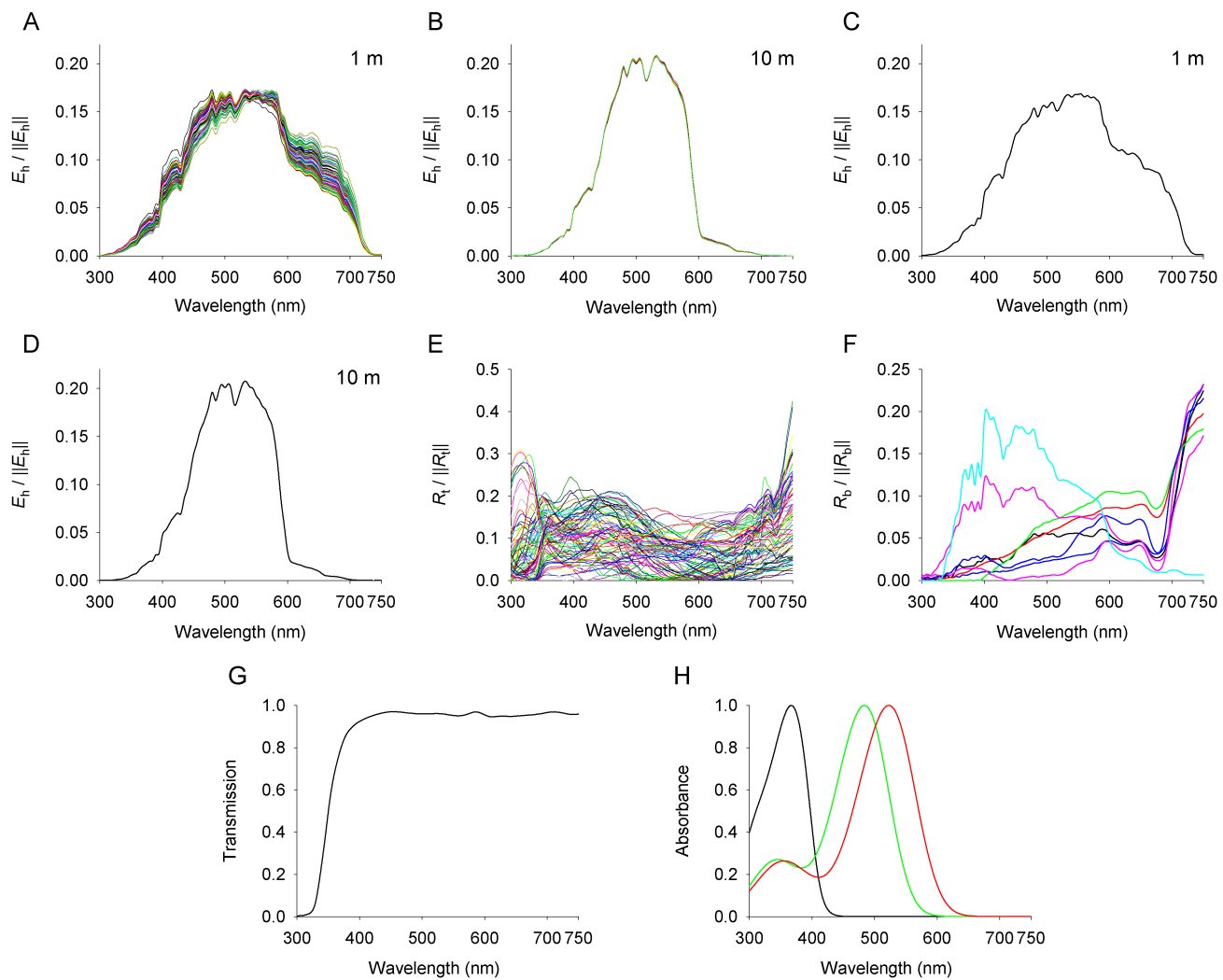

Supplement: Additional file 5 — Spectra used for chromatic contrast modelling. (A,B) A total of 100 spectra of sideward irradiance at a water depth of 1 m (A) and 10 m (B). These spectra were taken as the irradiance that illuminated the stimulus fish and the vertical rock substrate. (C,D) Mean spectral sideward irradiance at a depth of 1 m (C) and 10 m (D) that was taken as the irradiance that adapted the viewer fish eye. (E) Spectral reflectance of the body pattern of fish (n = 87). (F) Spectral reflectance of diverse rock substrates (n = 8). (G) Spectral transmission of the lens in Metriaclima zebra. (H) Spectral absorbance templates for visual pigments of A1 chromophore constructed based on the cone pigments typically found in adult M. zebra: a single cone-occupying pigment (SWS1, λmax = 368 nm) and two double cones-occupying pigments (Rh2b, λmax = 484 nm; Rh2a, λmax = 523 nm). For graphical presentation only, each of the spectra presented in (A-F) was normalized by its norm. [file 1741-7007-11-77-S5.pdf]
