# Supplementary material for: What has driven the evolution of multiple cone classes in visual systems: object contrast enhancement or light flicker elimination?
Source: BMC Biol. 2013 Jul 4;11:77. doi: 10.1186/1741-7007-11-77 (PMC3720213; doi:10.1186/1741-7007-11-77)
Supplement: Additional file 3 — Compilation of critical fusion frequency (CFF) and the frequency at which maximum contrast sensitivity is attained (Fmax, estimated as 15% of CFF) in fish. Frequencies are given in Hz. [file 1741-7007-11-77-S3.pdf]

Additional file 3. Compilation of critical fusion frequency (CFF) and the frequency at which maximum contrast sensitivity is attained (F_{max}, estimated as 15% of CFF) in fish. Frequencies are given in Hz. Additional references are provided below table.

Species	Order, Family	Common name	Average stimulus intensity						Adapt.	Temp. (ºC)	Habitat	Depth	Reference
			Dim		Bright		Natural						
			F_{max}	CFF	F_{max}	CFF	F_{max}	CFF					
Gymnothorax reticularis	Anguilliformes, Muraenidae	NA	0.9	6	2.4	16			Dark	20	Benthic	Deep	[1]
Leuresthes tenuis	Atheriniformes, Atherinopsinae	California grunion			9	60			Light	22	Benthopelagic	Shallow	[2]
Alepisaurus ferox	Aulopiformes, Alepisauridae	Lancetfish	10.5	70	13.2	88			Dark	NA	Benthopelagic	both	[3]
Carcharhinus acronotus	Carcharhiniformes, Carcharhinidae	Blacknose shark	2.4	16.0	2.7	18			Dark	25	Benthopelagic	both	[4]
Sphyrna lewini	Carcharhiniformes, Sphyrnidae	Scalloped hammerhead	3.8	25	4.1	27			Dark	25	Pelagic	both	[4]
Sphyrna tiburo	Carcharhiniformes, Sphyrnidae	Bonnethead shark	3.9	26	4.7	31			Dark	25	Benthopelagic	both	[4]
Carassius auratus	Cypriniformes, Cyprinidae	Goldfish	1.8	12	3	20			Dark	10	Benthopelagic	Shallow	[1]
Carassius auratus			1.5	10	3.6	24			Light	5			[5]
Carassius auratus			3	20	6.5	43			Light	15			[5]
Carassius auratus			5	33	10.1	67			Light	25			[5]
Carassius auratus			0.5		2				Dark	NA			[6]
Cyprinus carpio	Cypriniformes, Cyprinidae	Carp	0.9	6	2.7	18			Light	13	Benthopelagic	Shallow	[7]
Cyprinus carpio			3.8	25	5.9	39			Light	30			[7]
Heterodontus francisci	Heterodontiformes, Heterodontidae	Horned shark			2.7	18			Light	13	Benthic	Shallow	[2]
Mugil cephalus	Mugiliformes, Mugilidae	Flathead grey mullet			15	100			Light	25	Benthopelagic	Shallow	[2]
Platyrhinoidis triseriata	Myliobatiformes, Platyrhinidae	Thornback ray			6.8	45			Light	16	Benthic	Shallow	[2]

Species	Order, Family	Common name	Average stimulus intensity						Adapt.	Temp. (ºC)	Habitat	Depth	Reference
			Dim		Bright		Natural						
			F_{max}	CFF	F_{max}	CFF	F_{max}	CFF					
Urolophus fuscus	Myliobatiformes, Urolophidae	Round stingrays	0.7	4.4	0.9	6			Dark	20	Benthic	Shallow	[8]
Trachurus japonicus	Perciformes, Carangidae	Japanese jack mackerel	1.5	10	3.5	23			Dark	10	Pelagic	Deep	[9]
Lepomis Sp.	Perciformes, Centrarchidae	Sunfish	1.5	10	7.5	50			Dark	21	Benthopelagic	Shallow	[9]
Coryphaena hippurus	Perciformes, Coryphaenidae	Dolphinfish	4.1	27	13.2	88			Dark	NA	Pelagic	both	[9]
Lepidocybium flavobrunneum	Perciformes, Gempylidae	Escolar	0.3	2	3.3	22			Dark	NA	Benthopelagic	Deep	[1]
Tetrapturus audax	Perciformes, Istiophoridae	Striped marlin	4.5	30	8.6	57			Dark	NA	Pelagic	Deep	[10]
Halichoeres poecilopterus	Perciformes, Labridae	Multicolorfin rainbow fish	1.1	7	2	13			Dark	10	Benthic	Shallow	[3]
Morone saxatilis	Perciformes, Moronidae	Striped bass			11.3	75	7.5	50	Dark	20	Benthopelagic	Shallow	[3]
Pagothenia borchgrevinki	Perciformes, Nototheniidae	Bald notothen			2.1	14	1.2	8	Dark	0	Cryopelagic	Shallow	[3]
Trematomus bernacchi	Perciformes, Nototheniidae	Emerald rockcod			1.4	9	0.6	4	Dark	0	Cryobenthic	both	[1]
Trematomus centronotus	Perciformes, Nototheniidae	Sharp-spined notothenia			1.2	8	0.5	3	Dark	0	Cryobenthic	both	[11]
Trematomus hansoni	Perciformes, Nototheniidae	Striped rockcod			0.8	5	0.3	2	Dark	0	Cryopelagic	both	[11]
Pomatomus saltatrix	Perciformes, Pomatomidae	Bluefish			10.1	67	7.1	47	Dark	20	Pelagic	both	[11]
Rachycentron canadum	Perciformes, Rachycentridae	Cobia			9.8	65	8	53	Dark	20	Benthopelagic	both	[11]
Cynoscion nebulosus	Perciformes, Sciaenidae	Spotted weakfish			9	60	4.8	32	Dark	20	Benthopelagic	Shallow	[12]
Cynoscion regalis	Perciformes, Sciaenidae	Squeteague			6	40	4.2	28	Dark	20	Benthopelagic	Shallow	[12]
Leiostomus xanthurus	Perciformes, Sciaenidae	Spot croaker			8.1	54	4.4	29	Dark	20	Benthopelagic	both	[12]
Micropogonias undulatus	Perciformes, Sciaenidae	Atlantic croaker			8.9	59	5	33	Dark	20	Benthopelagic	both	[12]

Species	Order, Family	Common name	Average stimulus intensity						Adapt.	Temp. (ºC)	Habitat	Depth	Reference
			Dim		Bright		Natural						
			F_{max}	CFF	F_{max}	CFF	F_{max}	CFF					
Sciaenops ocellatus	Perciformes, Sciaenidae	Red drum			8.1	54	4.8	32	Dark	20	Benthopelagic	Shallow	[12]
Thunnus albacares	Perciformes, Scombridae	Yellowfin tuna			12	80			Light	25	Pelagic	both	[2]
Thunnus albacares					7.5	50			Dark	22			[13]
Thunnus albacares					2.7	18			Dark	15			[14]
Thunnus albacares					5.4	36			Dark	22			[14]
Thunnus maccoyii	Perciformes, Scombridae	Southern bluefin tuna	4.8	32	5.3	35			Dark	NA	Pelagic	Deep	[3]
Thunnus obesus	Perciformes, Scombridae	Bigeye tuna			5.4	36			Dark	22	Pelagic	both	[13]
Paralabrax nebulifer	Perciformes, Serranidae	Barred sand bass			7.5	50			Light	12	Benthic	both	[2]
Sillago japonica	Perciformes, Sillaginidae	Japanese whiting	1.4	9	3.3	22			Dark	20	Benthopelagic	Shallow	[1]
Pagrus major	Perciformes, Sparidae	Red seabream	1.7	11	2.4	16			Dark	20	Benthopelagic	both	[1]
Xiphias gladius	Perciformes, Xiphiidae	Swordfish	0.3	2	6	40			Dark	22	Pelagic	both	[13]
Paralichthys dentatus	Pleuronectiformes, Paralichthyidae	Summer flounder			7.8	52	5.1	34	Dark	20	Benthopelagic	both	[9]
Raja erinacea [a]	Rajiformes, Rajidae	Little skate	0.9	6	4.5	30			Dark/ Light	NA	Benthic	both	[15]
Rhinobatos productus	Rajiformes, Rhinobatidae	Guitarfish			4.5	30			Light	16	Benthic	Shallow	[2]
Salmo salar	Salmoniformes, Salmonidae	Atlantic salmon	2.4	16	7.2	48			Light	5	Benthopelagic	Shallow	[16]
Salmo salar			3.9	26	11.1	74			Light	15			[16]
Salmo salar			4.5	30	14.4	96			Light	25			[16]
Salvelinus fontinalis	Salmoniformes, Salmonidae	Brook trout	4.5	30	10.1	67			Light	NA	Benthopelagic	Shallow	[17]

Species	Order, Family	Common name	Average stimulus intensity						Adapt.	Temp. (ºC)	Habitat	Depth	Reference
			Dim		Bright		Natural						
			F_{max}	CFF	F_{max}	CFF	F_{max}	CFF					
Salvelinus fontinalis			1.5	10	3.2	21			Dark	NA			[17]
Sebastolobus altivelis	Scorpaeniformes, Sebastidae	Longspine thornyhead			1.5	10			Light	7	Benthic	Deep	[2]
Stephanolepis cirrhifer	Tetraodontiformes, Monacanthidae	Threadsail filefish	1.4	9	2.3	15			Dark	20	Benthopelagic	Shallow	[1]
Lagocephalus lunaris	Tetraodontiformes, Tetraodontidae	Lunartail puffer	3	20	2.4	16			Dark	20	Benthopelagic	both	[1]
Sphoeroides maculates	Tetraodontiformes, Tetraodontidae	Fugu	1.8	12	2.4	16			Dark	20	Benthopelagic	Shallow	[1]
Narke japonica	Torpediniformes, Narkidae	Japanese sleeper ray	0.6	4	0.9	6			Dark	20	Benthic	Shallow	[8]

[a] The Little skate, *Raja erinacea*, has a rod-only retina, and thus, was excluded from analysis.

Additional references

1. Kobayashi H: **A comparative study on electroretinogram in fish, with special reference to ecological aspects IV.** *J Shimoneseki Coll Fish* 1962, **11:**473-538.
2. Bullock TH, Hofmann MH, New JG, Nahm FK: **Dynamic properties of visual evoked potentials in the tectum of cartilaginous and bony fishes, with neuroethological implications.** *J Exp Zool* 1991, **5:**142-155.
3. Fritsches KA, Warrant E: **Differences in the visual capabilities of sea turtles and blue water fishes - implications for bycatch reduction.** In *Sea turtle and pelagic fish sensory biology: developing techniques to reduce sea turtle bycatch in longline fisheries* (eds Y. Swimmer and R. Brill). National Oceanic and Atmospheric Administration. 2006.
4. McComb DM, Frank TM, Hueter RE, Kajiura SM: **Temporal resolution and spectral sensitivity of the visual system of three coastal shark species from different light environments.** *Physiol Biochem Zool* 2010, **83:**299-307.
5. Hanyu I, Ali MA: **Flicker fusion frequency of electroretinogram in light-adapted goldfish at various temperatures.** *Science* 1963, **140:**662-663.
6. Bilotta J, Lynd FM, Powers MK: **Effects of mean luminance on goldfish temporal contrast sensitivity.** *Vision Res* 1998, **38:**55-59.
7. Tamura T, Hanyu I: **The flicker electroretinogram of the carp eye.** *Bull Jap Soc Sci Fish* 1959, **25:**624-631.
8. Kobayashi H: **A comparative study on electroretinogram in fish, with special reference to ecological aspects II. Electroretinogram of rays and skates, Batoidei, with reference to ecological aspects.** *J Shimoneseki Coll Fish* 1962, **11:**422-454.

9. Horodysky AZ, Brill RW, Warrant EJ, Musick JA, Latour RJ: **Comparative visual function in four piscivorous fishes inhabiting Chesapeake Bay.** *J Exp Biol* 2010, **213:**1751-1761.
10. Crozier WJ, Wolf E, Zerrahn-Wolf G: **On critical frequency and critical illumination for response to flickered light.** *J Gen Physiol* 1936, **20:**211-228.
11. Pankhurst NW, Montgomery JC: **Visual function in four Antarctic nototheniid fishes.** *J Exp Biol* 1989, **142:**311-324.
12. Horodysky AZ, Brill RW, Warrant EJ, Musick JA, Latour RJ: **Comparative visual function in five sciaenid fishes inhabiting Chesapeake Bay.** *J Exp Biol* 2008, **211:**3601-3612.
13. Fritsches KA, Brill RW, Warrant EJ: **Warm eyes provide superior vision in swordfishes.** *Curr Biol* 2005, **15:**55-58.
14. Fritsches KA, Warrant E: **New discoveries in visual performance of pelagic fishes.** Pelagic Fisheries Research Program; 2001, **6:** 1-3.
15. Green DG, Siegel IM: **Double branched flicker fusion curves from all-rod skate retina.** *Science* 1975, **188:**1120-1122.
16. Hanyu I, Ali MA: **Electroretinogram and its flicker fusion frequency at different temperatures in light-adapted Salmon (*Salmo salar*).** *J Cell Compar Physl* 1964, **63:**309-321.
17. Ali MA, Kobayashi H: **Electroretinogram - Flicker fusion frequency in albino trout.** *Experientia* 1968, **24:**454-455.
